# Deletion of the novel gene mother cell lysis X results in Cry1Ac encapsulation in the *Bacillus thuringiensis* HD73

**DOI:** 10.3389/fmicb.2022.951830

**Published:** 2022-08-09

**Authors:** Jiaojiao Wang, Qingyue Yu, Qi Peng, Leyla Slamti, Ruibin Zhang, Shuo Hou, Didier Lereclus, Fuping Song

**Affiliations:** ^1^State Key Laboratory for Biology of Plant Diseases and Insect Pests, Institute of Plant Protection, Chinese Academy of Agricultural Sciences, Beijing, China; ^2^Université Paris-Saclay, INRAE, AgroParisTech, Micalis Institute, Jouy-en-Josas, France; ^3^Shenzhen Branch, Guangdong Laboratory of Lingnan Modern Agriculture, Genome Analysis Laboratory of the Ministry of Agriculture and Rural Affairs, Agricultural Genomics Institute at Shenzhen, Chinese Academy of Agricultural Sciences, Shenzhen, China

**Keywords:** *Bacillus thuringiensis*, mother cell lysis, cell wall lytic enzyme C, Cry1Ac encapsulation, transcriptional regulation

## Abstract

The novel protein MclX (mother cell lysis X) in *Bacillus thuringiensis* subsp. *kurstaki* strain HD73 (*B. thuringiensis* HD73) was characterized in this work. MclX has no known domain and its gene deletion in HD73 resulted in Cry1Ac encapsulation in the mother cell and did not influence Cry1Ac protein production or insecticidal activity. *In vitro* cell wall hydrolysis experiments showed that MclX cannot hydrolyze the cell wall. In *mclX* deletion mutants, the expression of *cwlC* (which encodes a key cell wall hydrolase) was significantly decreased, as shown by the β-galactosidase activity assay. MclX cannot directly bind to the *cwlC* promoter, based on the electrophoretic mobility shift assay (EMSA). The *cwlC* was reported to be regulated by σ^K^ and GerE. However, the transcriptional activities of *sigK* and *gerE* showed no difference between HD73 and the *mclX* deletion mutant. It is indicated that MclX influenced *cwlC* expression independently of σ^K^ or GerE, through a new pathway to regulate *cwlC* expression. *mclX* deletion could be a new approach for insecticidal protein encapsulation in *Bacillus thuringiensis*.

## Introduction

*Bacillus thuringiensis* is a microbial insecticide used worldwide in agriculture (Jouzani et al., 2017). The most important feature of *B. thuringiensis* is the formation of spores and the production of insecticidal parasporal crystals. When *B. thuringiensis* grown to mature, the spore and crystal protein was released from mother cell. The crystal protein is toxic to Lepidoptera, Coleoptera, Diptera and other agricultural pests (Beegle and Yamamoto, 1992; Jouzani et al., 2017). However, the insecticidal activity of crystal protein was frequently reduced by environmental factors such as sunlight exposure (Myasnik et al., 2001; Zogo et al., 2019). Encapsulation of crystal proteins by physicochemical, mechanical, or molecular biological techniques can protect their activity from sunlight exposure (Lereclus et al., 1995; Manasherob et al., 2002; Jallouli et al., 2014; Chen et al., 2018; de Oliveira et al., 2021).

Encapsulation of crystal protein by physicochemical and mechanical techniques has some advantages and disadvantages. Encapsulation by extrusion is simple to use but low productivity, encapsulation by fluidized bed is high productivity but difficult to control, encapsulation by electrospinning is easy to expand production but the media may be toxic (de Oliveira et al., 2021). Crystal protein encapsulation in mother cell by molecular biological techniques was proved to be a good strategy to improve Cry protein stability under ultraviolet (UV) radiation (Sanchis et al., 1999; Zhou et al., 2014). The mother cell wall can protect Cry protein from inactivation by UV radiation. Deletion of *sigK* led to blocking mother cell lysis, encapsulating crystal proteins within the mother cell wall. This technology not only constructed spore-free strain, which had no pollution to the environment and no competition with wild *B. thuringiensis* strains, but also improved the UV resistance of crystal proteins in the field condition (Sanchis et al., 1999). Encapsulation Cry1Ba in the mother cell of *sigK* mutant also increasing UV radiation resistance (Zhou et al., 2014). However, some *cry* genes are regulated by σ^K^, and *cry* gene expressions were reduced in the *sigK* deletion mutant (Adams et al., 1991; Bravo et al., 1996).

Cell wall hydrolases are important in cell wall metabolism, they are classified as glycosidases and peptidases. Glycosidases (glucosaminidase, muramidase, and lytic transglycosylase) and peptidases (amidase, endopeptidase, and carboxypeptidase) cleave different sites of cell wall peptidoglycan (Do et al., 2020a). In *B. subtilis*, cell wall hydrolases involved in mother cell lysis (CwlB, CwlH, CwlC) are amidases with functional redundancy. Single amidase gene deletion did not affect mother cell lysis, while *cwlB cwlH cwlC* deletion mutant significant reduced mother cell lysis (Nugroho et al., 1999). In *B. thuringiensis*, CwlB and CwlC show low sequence identities with *B. subtilis* hydrolases. Deletion of the *cwlB* gene can delay lysis of mother cells (Yang et al., 2013). Deletion of the *cwlC* can block the lysis of mother cells without influence on Cry1Ac production (Chen et al., 2018). As the essential hydrolase in mother cell lysis, CwlC was reported to be regulated by σ^K^ and GerE in *B. thuringiensis* (Chen et al., 2018). No other genes have been reported to be deleted for encapsulation crystal protein except *sigK* and *cwlC*.

In this work, we identified a hypothetical gene whose deletion resulted in Cry1Ac encapsulation in the mother cell. The mother cell did not lysis because deletion of this hypothetical gene reduced the *cwlC* expression. This discovery of the regulatory mechanism of *cwlC* is independent from the regulatory pathway of σ^K^ or GerE. Studies on this new gene may contribute to a deeply understanding of *cwlC* expression. The deletion of this new gene encapsulated Cry1Ac in the mother cell with no effect on sporulation frequency, Cry1Ac protein production, or insecticidal activity. This could be a potential new approach for Cry protein encapsulation by molecular biology techniques.

## Materials and methods

### Bacterial strains, growth conditions, and plasmids

Tables showed the strains (Supplementary Table 1), primers (Supplementary Table 2), and plasmids (Supplementary Table 3) used in this work. The *B. thuringiensis* subsp. *kurstaki* strain HD73 (GenBank Accession Number: NC_020238.1) was used for transformations (Du and Nickerson, 1996; Liu et al., 2017). All *B. thuringiensis* strains were cultured in SSM (Schaeffer’s sporulation medium) (Schaeffer et al., 1965) or LB (Luria-Bertani) medium under 30°C, with 100 μg/ml Kan (Kanamycin) or 5 μg/ml Ery (Erythromycin) if needed. The *E*. *coli* strain TG1 was used for constructing the vector (Hoffmann et al., 2015). The *E*. *coli* ET12567 was used for extracting non-methylated vectors on a large-scale and transforming into *B. thuringiensis*. All *E*. *coli* strains were grown in LB medium at 37°C, with 100 μg/ml Amp (Ampicillin) if needed.

### DNA manipulation and transformation

The *EasyPure* Plasmid MiniPrep Kit (Transgen, Beijing, China) was used to extract vectors from *E. coli* cells listed in Supplementary Table 3. The PrimeSTAR Max DNA Polymerase (Takara, Beijing, China) was used to perform PCR, using a Mastercycler X50 (Eppendorf). Primers were synthesized in a company (Sangon, China). The *EasyPure* Quick Gel Extraction Kit (Transgen, Beijing, China) was used to purified DNA fragments, the Seamless Assembly Cloning Kit (Clonesmarter, United States) was used to connect DNA fragment and the linearized vector. Plasmid was introduced into the strain HD73 by electroporation (GenePulser Xcell, Bio-Rad).

### Construction of the mother cell lysis X deletion mutant strain HD (Δ*mclX*)

To delete the *mclX* gene in the HD73 genome, pMAD (Arnaud et al., 2004) (a temperature-sensitive suicide plasmid) with the *mclX* mutation box was constructed. The *mclX* mutation box was amplified as follows. A 725-bp fragment upstream of *mclX* (*mclX* fragment A) which contains a 12-bp overlap with the *mclX* 5′ end, was amplified by PCR, and *mclX*-1 and *mclX*-2 were used as primers. A 677-bp fragment downstream of *mclX* (*mclX* fragment B) which starts 4-bp away from *mclX* 3′ end, was amplified by PCR, and *mclX*-5 and *mclX*-6 were used as primers. A 1,473-bp fragment of the *kan* resistance gene was amplified by PCR, and *mclX*-3 and *mclX*-4 were used as primers. The overlapping PCR was used in amplifying the *mclX* mutation box (*mclX* fragment A, *kan* fragment, and *mclX* fragment B), using *mclX*-1 and *mclX*-6 as primers, and the resulting fragment of the *mclX* mutation box was inserted into the pMAD plasmid. Then, the recombinant plasmid was introduced into HD73. The *mclX* mutation box allelic replacement was performed as reported (Yang et al., 2013). The *mclX* ORF (12–663) was replaced by *kan* fragment which detected by PCR with primers *mclX*-1 and *mclX*-6.

### Construction of the complemented strain

To construct the complemented strain HD (Δ*mclX:mclX*), the *mclX* promoter and open reading frame (P*mclX*-*mclX*, 998-bp) were amplified by PCR, using HF*mclX*-F and HF*mclX*-R as primers. The P*mclX*-*mclX* fragment was inserted into the pHT315 plasmid (Arantes and Lereclus, 1991) to generate pHTHF*mclX*. The plasmid pHTHF*mclX* was introduced into HD (Δ*mclX*) to generate complemented strain HD (Δ*mclX*:*mclX*).

### Construction of a P*mclX-lacZ* fusion strain

To analyze the transcriptional activity of the *mclX* promoter, a 335-bp fragment upstream of the *mclX* ATG start codon was amplified by PCR, using P*mclX*-F and P*mclX*-R as primers. The P*mclX* fragment was inserted into the pHT304-18Z plasmid (Hervé and Lereclus, 1994) carrying the *lacZ* gene. The recombinant plasmid pHTP*mclX* was introduced into the HD73 strain, mutant strain HD (Δ*sigK*) or HD (Δ*gerE*).

### Construction of a mother cell lysis X-His fusion protein

To express the MclX protein in an *E. coli* strain, an *mclX* gene fragment without a termination codon was amplified by PCR, using MclX-F and MclX-R as primers. The resulting fragment was inserted into the pET21b plasmid to generate pET*mclX* with a C-terminal His tag. The plasmid pET*mclX* was transformed into the BL21 strain for expressing the MclX-His protein.

### Expression and purification of mother cell lysis X-His protein

The method of protein expression and purification was previously described (Zhang et al., 2020). A BL21(pET*mclX*) strain was grown in LB liquid medium until reaching an OD_600_ of 0.7. Then, 1 mM isopropyl-beta-D-thiogalactoside (Solarbio, Beijing, China) was added to induce MclX-His expression, and bacteria were harvested after overnight growth at 18°C and sonicated (CP750, Cole-Parmer). The supernatant was loaded onto a 2-ml suspension of Ni Sepharose 6 Fast Flow (GE Healthcare, Sweden) with wash buffer (25 mM Tris-HCl, 50 mM imidazole, 0.5 M NaCl, pH 8.0) and elution buffer (25 mM Tris-HCl, 250 mM imidazole, 0.5 M NaCl, pH 8.0). The purified MclX-His protein was analyzed by 10% SDS–PAGE (Mini protein III, Bio-rad).

### β-galactosidase assays

The method of the β-galactosidase activity assay was previously described (Miller, 1972). The error bars represent three independent experiment results.

### Sporulation frequency analysis

Liquid samples (50-ml) of HD73 and HD (Δ*mclX*) were harvested at T24 (24 h after the exponential phase ended). Heated samples (under 65°C for 20 min) and unheated samples were diluted in a gradient, coated on the LB agar plates. Then the number of grown colonies in heated and unheated sample plates was counted. The sporulation frequency is the clone number ratio of the heated sample to the unheated sample. The error bars represent three independent experiment results.

### Cry1Ac protein production

The method was performed as previous described (Zhang et al., 2018). The wild-type strain HD73 and the strain HD (Δ*mclX*) were harvested at T24 and lyophilized into powder, same quality of samples was taken and determined by 10% SDS–PAGE (Bio-rad). The results of three independent experiments are consistent.

### Cell wall preparation and hydrolysis

For cell wall preparation and hydrolysis, method was previously described (Yang et al., 2013; Chen et al., 2018). CwlC added to cell wall was used as a positive control. The cell wall itself was used as a negative control, and the weight of MclX was the same as that of CwlC. The results of three independent experiments are consistent.

### Bioassay of insecticidal activity

A bioassay was carried out as previous described (Chen et al., 2018). Each test was conducted with three independent cultures replicated.

### Western blot analysis

Cell lysates (the equivalent of 2 ml at the time point) were suspended in 600 μl of Tris buffer (pH 8.0) and analyzed by 10% SDS–PAGE (Bio-rad). Anti-CwlC was generated by a company (Beijing Protein Innovation Inc., China), and the second antibody (HRP-conjugated goat anti-mouse IgG) was bought (CWBiotech, Beijing, China). The results of three independent experiments are consistent.

### Electrophoretic mobility shift assays

EMSA (Electrophoretic mobility shift assay) was carried out as previous described (Shen et al., 2021). The *cwlC* promoter was labeled with 6-carboxyfluorescein (FAM) (Supplementary Table 3). The results of three independent experiments are consistent.

## Results

### Deletion of the hypothetical gene HD73_*RS12920* can encapsulate Cry1Ac in mother cell

To search for key genes involved in mother cell lysis, T7 transcriptome data (Tn stands for n hours after the exponential phase ended) of the strain HD73 was analyzed (Peng et al., 2015). T7 stands for the late sporulation stage, at which the exosporium genes of the spore coat begin to be transcribed (Peng et al., 2016). Transcription of *cwlC* had not started at T7, which means that mother cell lysis also had not started. Highly expressed hypothetical genes were screened (Supplementary Table 4) and genetic deletion mutants were constructed.

Fortunately, deletion of a hypothetical gene showed a phenotype in which the mother cell did not lyse. This hypothetical gene is HD73_*RS12920* (which encodes a protein in NCBI with the reference sequence WP_000101499.1). HD73_*RS12920* is 663-bp in length and has no known domains. According to the deletion mutant phenotype, which displayed no mother cell lysis, HD73_*RS12920* was named *mclX* (mother cell lysis X).

**FIGURE 1:**
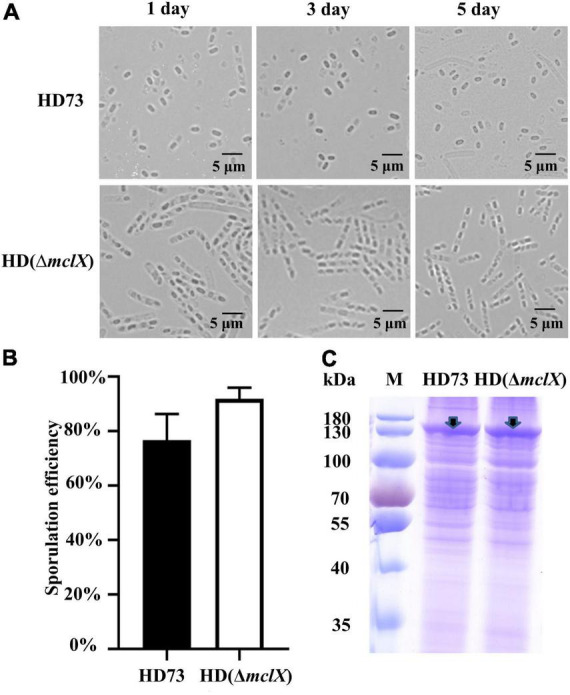
Phenotype analysis of the HD (Δ*mclX*) strain. **(A)** Phenotype of the wild-type strain HD73 and HD (Δ*mclX*) at 1, 3, and 5 days after the bacterial growth reached the end of the exponential phase. Scale bars, 5 μm; **(B)** sporulation frequency of the wild-type strain HD73 and HD (Δ*mclX*). Error bars show the standard error of the mean; **(C)** Cry1Ac protein production in the wild-type strain HD73 and HD (Δ*mclX*). Cry1Ac production bands are marked by arrows. Lane M, protein marker (26616).

In the mc*lX* deletion mutant HD (Δ*mclX*) (method described in “Materials and Methods”), the mother cell did not lyse, while the mother cells of wild-type strain HD73 lysed at 1, 3, and 5 days after the bacterial growth reached the end of the exponential phase (Figure 1A). There was no difference in vegetative growth between the wild-type strain HD73 and HD (Δ*mclX*) (Supplementary Figure 1). To further demonstrate the role of MclX in mother cell lysis, we constructed a genetically complemented strain HD (Δ*mclX:mclX*), as described in section “Materials and methods.” In the mother cell lysis stage, most wild-type and HD (Δ*mclX*:*mclX*) mother cells have lysed (Supplementary Figure 2). The results showed that MclX played an important role in mother cell lysis.

There were no differences between the wild-type strain HD73 and HD (Δ*mclX*) in sporulation frequency or Cry1Ac protein production at T24 (Figures 1B,C). The second instar larva of *Plutella xylostella* was used to determine the insecticidal activity of the wild-type strain HD73 and HD (Δ*mclX*). *P. xylostella* larvae were fed prepared cabbages treated with a mixture of spores and crystals of the wild-type strain or the HD (Δ*mclX*) strain. The LC_50_ of the wild-type strain HD73 was 8.89 μg/ml and the LC_50_ of the HD (Δ*mclX*) strain was 10.2 μg/ml, displaying no significant differences (Table 1). Therefore, the deletion of the *mclX* encapsulated Cry1Ac in HD73 mother cells, without effect on sporulation frequency, Cry1Ac protein production, or insecticidal activity.

**TABLE 1:** Insecticidal activities of *B. thuringiensis* strains against *Plutella xylostella.*

Strain	LC_50_ (μ g of protein/ml)	95% confidence interval
HD73	8.89	6.95–13.96
HD(Δ*mclX*)	10.2	7.81–16.01

### Transcriptional regulation of the mother cell lysis X gene

**FIGURE 2:**
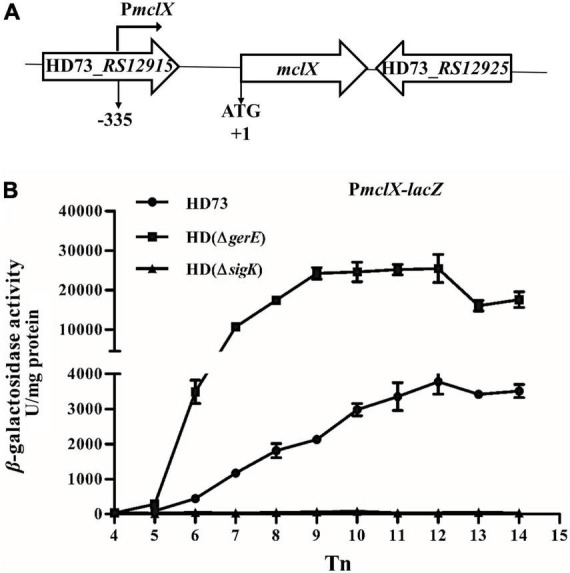
*mclX* transcription activity. **(A)** Gene map of the *RS12915*-*RS12925*; **(B)** *mclX* transcription activities in the wild-type strain HD73, HD (Δ*gerE*), and HD (Δ*sigK*) (HD73-P*mclX*, circles; HDΔ*gerE*-P*mclX*, squares; HDΔ*sigK*-P*mclX*, triangles). Tn means n hours after the exponential phase ended. Error bars show standard error of the mean.

MclX has no known domain and a molecular size of 25.6 kDa. Secondary structure alignment (PSIPRED)^1^ revealed the presence of several α-helixes and β-strands in MclX (Supplementary Figure 3). In the *B. thuringiensis* HD73 genome, *mclX* has an upstream gene, *RS12915*, which encodes a YozE family protein, and a downstream gene, *RS12925*, which encodes a hypothetical protein (Figure 2A). In the *B. cereus* group, MclX is very conserved (Supplementary Figure 4). *Streptococcus pneumoniae*, *Bacillus subtilis*, and *Rhodococcus qingshengii*, have a MclX homologous protein with 100, 62, and 65% similarity, respectively.

The promoter region of the *mclX* gene (335-bp upstream of the *mclX* ATG start codon) was fused to the *lacZ* gene, and transformed into HD (Δ*sigK*), HD (Δ*gerE*), and wild-type HD73. In HD73, the transcription of P*mclX* initiated at T6 and reached a peak at T12 (Figure 2B, circles). In HD (Δ*sigK*), the transcriptional activity of P*mclX* was completely abolished (Figure 2B, triangles). In HD (Δ*gerE*), P*mclX* transcriptional activity was increased compared to that in wild-type strain HD73 (Figure 2B, squares). All above results demonstrate that *mclX* was highly expressed in the late sporulation stage, controlled by σ^K^, and negatively regulated by GerE.

### Mother cell lysis X cannot hydrolyze the cell wall *in vitro*

HD (Δ*mclX*) displayed no mother cell lysis. MclX has no known domains, including DNA binding domains or any functional domains. Regarding its role in mother cell lysis, MclX may be an essential cell wall hydrolase or it may affect the function of the cell wall hydrolase CwlC.

**FIGURE 3:**
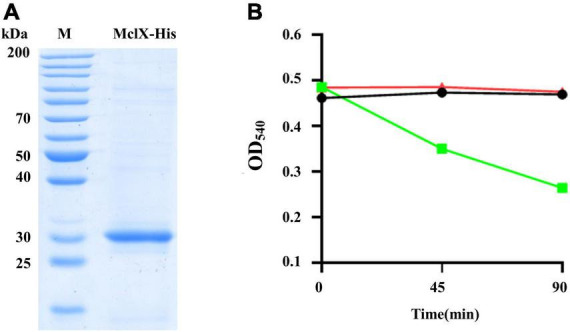
Characterization of the MclX protein. **(A)** MclX protein expressed in the recombinant strain *E*. *coli* BL21 (pETMclX) by SDS–PAGE analysis. M, protein marker, 26614; **(B)** purified MclX protein was unable to hydrolyze the wild-type strain HD73 cell wall. The strain HD73 cell wall was mixed with MclX protein (triangles). CwlC protein (known cell wall hydrolase) plus cell wall was used as a positive control (squares), and the cell wall itself was used as a negative control (circles).

To test whether MclX could be a cell wall hydrolase, the MclX protein hydrolyzed cell wall was analyzed *in vitro*. The MclX-His protein was expressed and analyzed by SDS–PAGE, its molecular weight was approximately 30 kDa (Figure 3A). The MclX-His protein and the wide-type strain HD73 cell wall were incubated together. The optical density at 540 nm (OD_540_) showed cell wall turbidity. CwlC is a known hydrolase that can reduce the cell wall turbidity (Figure 3B, squares). However, there was no reduction in the OD_540_ values of MclX protein-containing cell wall (Figure 3B), suggesting that MclX cannot hydrolyze the cell wall *in vitro*.

### Mother cell lysis X gene deletion decreases *cwlC* expression

Previous research indicated that *cwlC* is controlled by σ^K^ and positively regulated by GerE (Chen et al., 2018). Since HD (Δ*mclX*) exhibited a similar phenotype to HD (Δ*cwlC*), whether the deletion of the *mclX* affected the transcriptional activity or protein expression of CwlC was analyzed.

**FIGURE 4:**
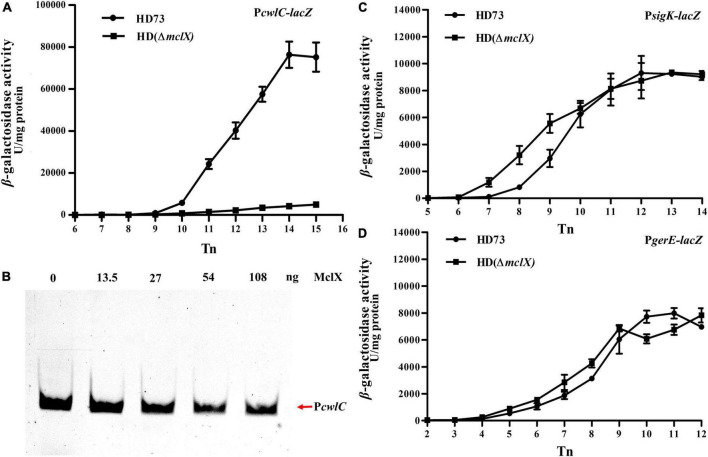
MclX indirectly affects the transcription of *cwlC*. **(A)** *cwlC* promoter activities in the wild-type strain HD73 and HD (Δ*mclX*) (HD73-P*cwlC*, circles; HDΔ*mclX*-P*cwlC*, squares); **(B)** the *cwlC* promoter was unable to interact with the MclX protein based on EMSA. Lane 1, 1 ng FAM-labeled P*cwlC* probe; lanes 2–5, 1 ng labeled probe with an increasing mass of purified MclX protein; **(C)** *sigK* promoter activities in the wild-type strain HD73 and HD (Δ*mclX*) (HD73-P*sigK*, circles; HDΔ*mclX*-P*sigK*, squares); **(D)** *gerE* promoter activities in the wild-type strain HD73 and HD (Δ*mclX*) (HD73-P*gerE*, circles; HDΔ*mclX*-P*gerE*, squares). Error bars show standard error of the mean.

The transcriptional activity of *cwlC* was investigated in HD (Δ*mclX*). β-galactosidase activity assays indicated that *cwlC* expression significantly decreased in HD (Δ*mclX*), compared to that in wild-type strain (Figure 4A). Whether MclX could directly bind to the *cwlC* promoter was tested by EMSA. MclX was found to be unable to directly bind to the *cwlC* promoter (Figure 4B). It is speculated that *mclX* deletion might affect *cwlC* transcription levels by affecting transcriptional activities or protein functions of σ^K^ and GerE. To investigate this, the transcriptional activities of *sigK* and *gerE* in HD (Δ*mclX*) and the wild-type strain were analyzed, showing no differences (Figures 4C,D). The protein functions of σ^K^ and GerE in HD (Δ*mclX*) were detected. *bxpB*, which was found to be regulated by σ^K^ and GerE (Peng et al., 2016), showed no differences in transcription between the wild-type strain and HD (Δ*mclX*) (Supplementary Figure 5). Thus, MclX indirectly affected *cwlC* transcriptional activity, not by affecting σ^K^ or GerE.

**FIGURE 5:**
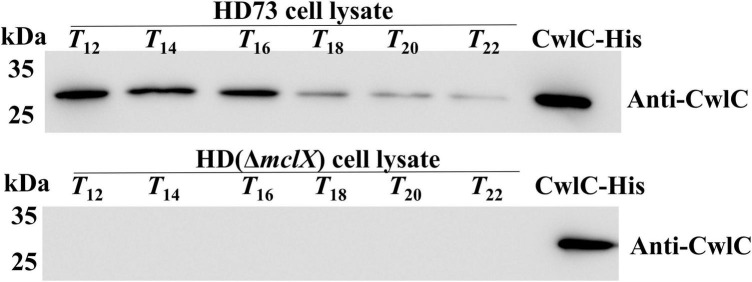
CwlC was determined by Western blot in wild-type HD73 and HD (Δ*mclX*) lysates. The same volumes were taken at the time points, and the same loading volumes were analyzed. CwlC-His served as positive control.

Moreover, CwlC protein production was investigated in HD (Δ*mclX*). In HD (Δ*mclX*) cell lysate, CwlC protein was barely visible in the anti-CwlC immunoblot, whereas significant amounts of CwlC protein were produced in the wild-type strain cell lysate (Figure 5), with the same bacterial volumes taken at the same time point and using the same loading volumes.

Altogether, CwlC expression was dramatically decreased and barely detectable in HD (Δ*mclX*).

## Discussion

In this work, the novel gene *mclX* was characterized, whose deletion resulted in Cry1Ac encapsulation in the mother cell of the HD73 strain (Figure 1A). MclX was unable to hydrolyze the cell wall *in vitro* (Figure 3B), and it is a newly identified mother cell lysis-associated protein which is not an amidase. MclX can be found not only in the *B. cereus* group but also in some *S. pneumoniae* strains (Supplementary Figure 4). *S. pneumoniae* is a Gram-positive human pathogen leading to global health problems (Engholm et al., 2017). Its MclX homologous protein shares 100% similarity with that of HD73, and the role of MclX in *S. pneumoniae* cell lysis maybe very interesting and warrants further studies.

*cwlC* encodes a key cell wall hydrolase, and its deletion resulted in Cry1Ac encapsulation in the HD73 strain. Only a few reports about the regulation of *cwlC*, while the regulation mechanisms of some other hydrolases have been reported (Do et al., 2020a). Transcription of the hydrolases *lytE* and *cwlO* is regulated by the WalKR signal transduction pathway in *B. subtilis* (Dobihal et al., 2019). The endopeptidase EagA is regulated through bacterial second messenger molecule c-di-GMP signaling in *Erwinia amylovora* (Kharadi and Sundin, 2020). Protein interactions with hydrolases can activate (Uehara et al., 2009, 2014; Yang et al., 2011; Domínguez-Cuevas et al., 2013; Meisner et al., 2013; Do et al., 2020b) or inhibit hydrolase activities (Clarke et al., 2010). The mother cell of the HD (Δ*mclX*) strain did not lyse because the *cwlC* expression decreased (Figure 4A). The expression of *mclX* was detected earlier than that of *cwlC* (Figures 2B, 4A), although MclX cannot directly bind the *cwlC* promoter (Figure 4B). The way in which MclX affects the transcriptional activity of *cwlC* remains unknown. MclX may influence *cwlC* transcription by affecting other factors, and not by affecting σ^K^ or GerE (Figures 4C,D). The function of MclX requires further research, to gain a better understanding of the regulatory mechanism of *cwlC*.

The HD (Δ*mclX*) strain displayed no mother cell lysis and did not have an altered sporulation frequency, Cry1Ac protein production, or insecticidal activity (Figure 1 and Table 1). *mclX* deletion could be a new biotechnological approach in the encapsulation of Cry proteins resistant to adverse environmental factors. The deletion of *cwlC* or *sigK* in the *Bacillus thuringiensis* var. *israelensis* (Bti) strain did not block mother cell lysis (Xu et al., 2020; Huang et al., 2022), because other cell wall hydrolases, like CwlE, are involved in mother cell lysis in Bti (Huang et al., 2022). However, the deletion of the *mclX* homolog gene in Bti also did not block mother cell lysis (Supplementary Figure 6). In comparison to the deletion of *cwlC* in Bti, the deletion of the *mclX* homolog gene in Bti led to more effective delays in mother cell lysis (Supplementary Figure 6). Thus, insecticidal protein encapsulation in mother cells requires targeted strategies in different *B. thuringiensis* strains.

## Conclusion

Deletion of *mclX* provides a novel approach for encapsulation of Cry protein in *B. thuringiensis*. MclX has no known domains and it is a key protein in the mother cell lysis of *B. thuringiensis* strain HD73. It is a discovery of blocking mother cell lysis which not cause by cell wall hydrolases. This strategy could be utilized to genetically modify the *B. thuringiensis* products for enhancing UV resistance.

## Data Availability statement

The original contributions presented in this study are included in the article/Supplementary material, further inquiries can be directed to the corresponding author.

## Author contributions

JW and QY conducted the experiments, analyzed the data, and produced the data displays. FS designed the research. QY wrote the manuscript. LS, DL, and QP edited the manuscript. RZ, SH, and QP proposed amendments. FS and QP funded the research. All authors contributed to the article and approved the submitted version.

## Footnotes

1
http://bioinf.cs.ucl.ac.uk/psipred/
