# Supplementary material for: Deletion of the novel gene mother cell lysis X results in Cry1Ac encapsulation in the Bacillus thuringiensis HD73
Source: Front Microbiol. 2022 Aug 9;13:951830. doi: 10.3389/fmicb.2022.951830 (PMC9397120; doi:10.3389/fmicb.2022.951830)
Supplement: Supplementary file 1 [file Data_Sheet_1.PDF]

Supplementary Material

Supplementary Figures

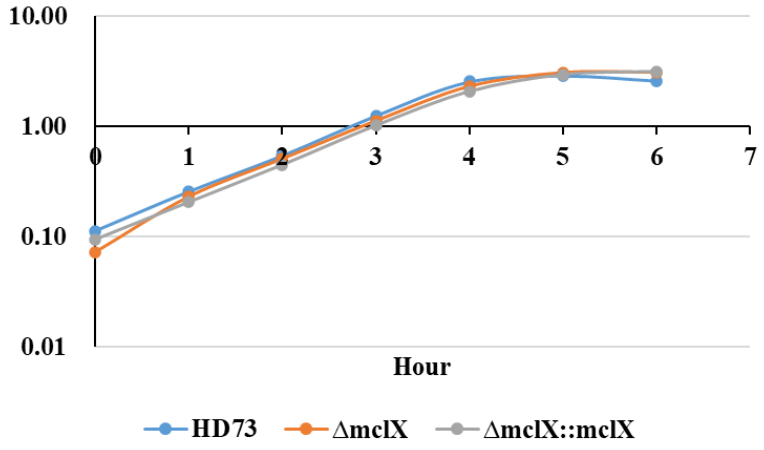

Supplementary Figure S1. Vegetative growth rates of the wild-type strain HD73, the mutant strain HD (Δ*mclX*) and the genetically complemented strain HD (Δ*mclX::mclX*) when the strains were grown in SSM medium at 30 °C.

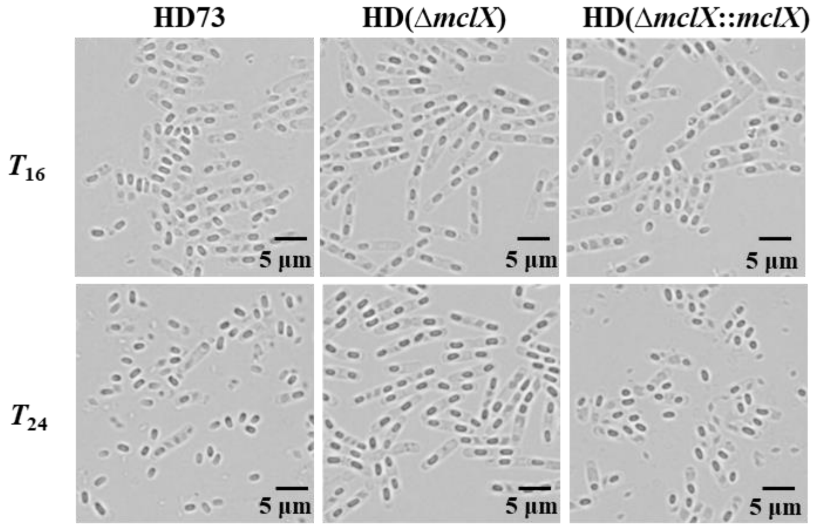

Supplementary Figure S2. The mother cell lysis of the wild-type strain HD73, the mutant strain HD (Δ*mclX*) and the genetically complemented strain HD (Δ*mclX::mclX*). Scale bars, 5 µm.

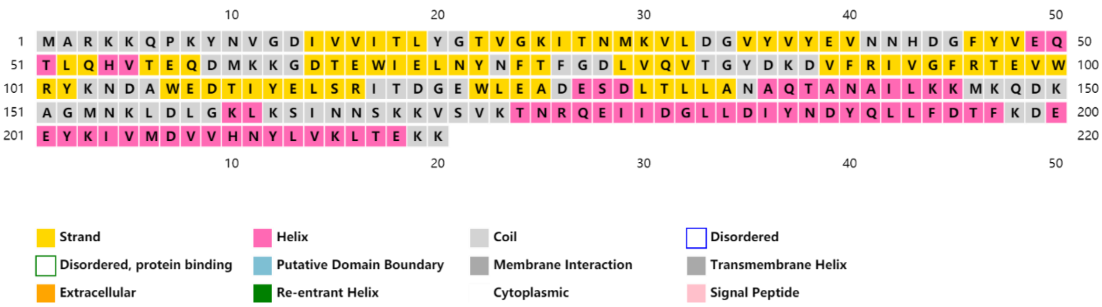

Supplementary Figure S3. PSIPRED predicted the secondary structure of the MclX protein.

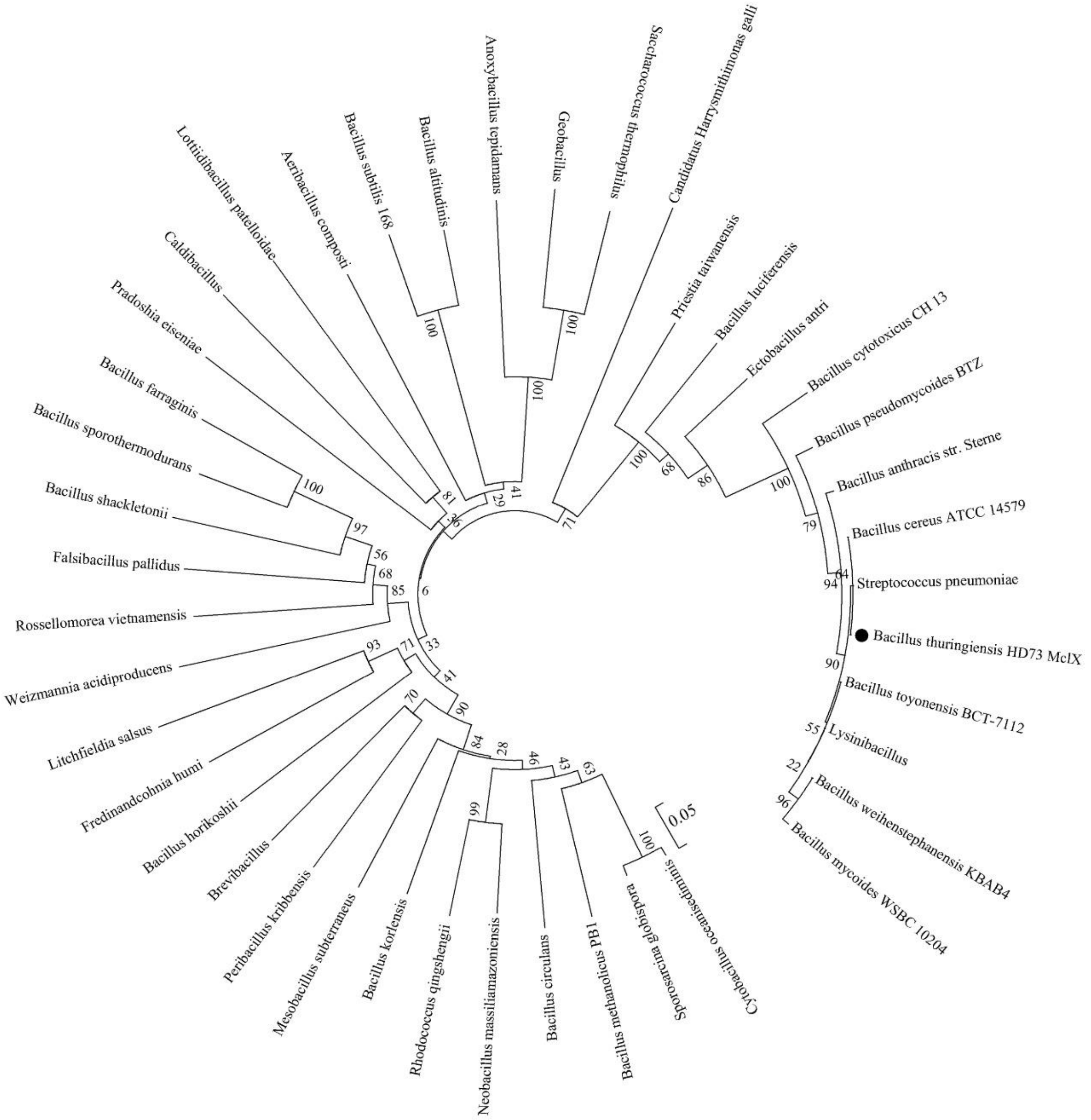

Supplementary Figure S4. Evolutionary relationship analysis of MclX. Homologous proteins with similarity greater than 50% were selected for analysis. A neighbor-joining tree was built using MEGA 5.0 software, p-distance method. In the *B. cereus* group, MclX is very conserved.

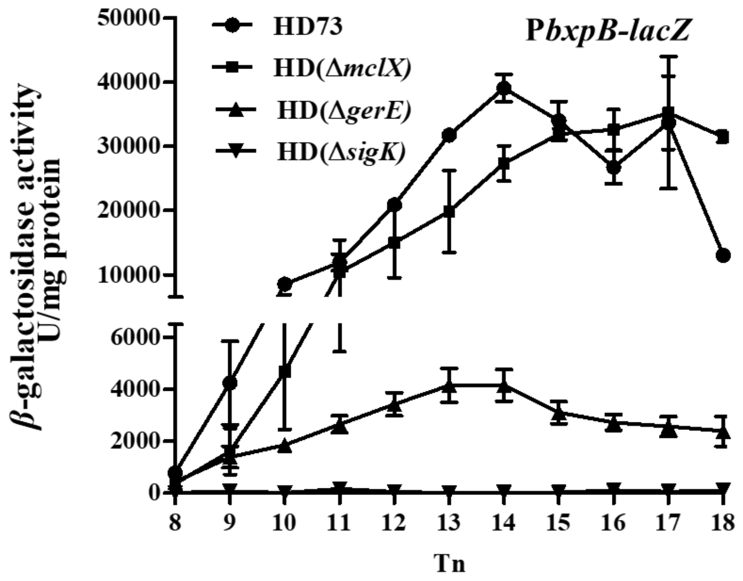

Supplementary Figure S5. Transcription activity analysis of *bxpB* gene in *B. thuringiensis*. *β*-galactosidase activity of *bxpB* gene in the wild-type strain HD73, HD (Δ*mclX*), HD (Δ*gerE*) and HD (Δ*sigK*). *bxpB* is regulated by GerE and σ^K. Tn, n hours after T0 (the end of the exponential growth phase). Each value represents the mean of three independent replicates. Error bars show standard deviations.

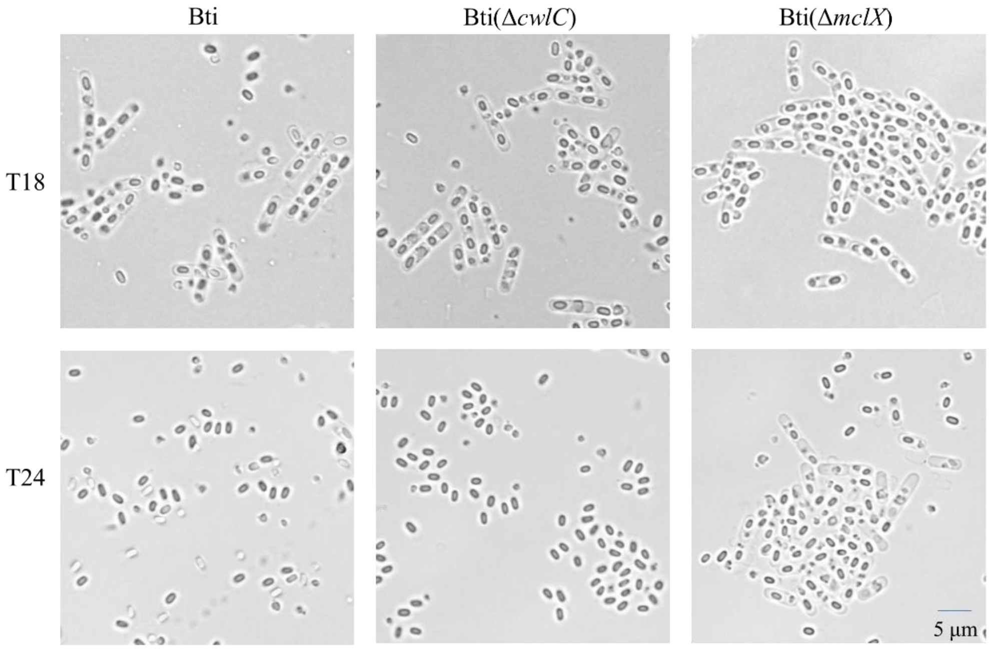

Supplementary Figure S6. The mother cell lysis of the wild-type strain Bti, Bti (Δ*cwlC*) and Bti (Δ*mclX*). Scale bars, 5 μm.
