# Supplementary material for: Deletion of the novel gene mother cell lysis X results in Cry1Ac encapsulation in the Bacillus thuringiensis HD73
Source: Front Microbiol. 2022 Aug 9;13:951830. doi: 10.3389/fmicb.2022.951830 (PMC9397120; doi:10.3389/fmicb.2022.951830)
Supplement: Supplementary file 2 [file Data_Sheet_2.docx]

Supplementary Material

## Supplementary Tables

**Supplementary Table S1.** Strains used in this study

| Strain | Characteristic(s) | Reference(s) or source |
| --- | --- | --- |
| *E*. *coli* strains |  |  |
| TG1 | Δ(*lac*-*proAB*) *supE* *thi hsd*-*5*(F’ *traD36 proA^+^ pro B^+^ lacl^q^ lacZ*ΔM15) | (Wang et al., 2009) |
| BL21(DE3) | Fˉ *dcm* *ompT* *hsdS*(r_B_ˉm_B_ˉ) *galλ*(DE3) | (Foster, 1992) |
| BL21(pET*mclX*) | BL21(DE3) with pET*mclX* plasmid | This study |
| SCS110 | Fˉ *dam-13::Tn9 dcm-6 hsdM-hsdR recF143 zjj-202::Tn10 galK2 galT22 ara14 pacY1 xyl-5 leuB6 thi-1* | (Wang et al., 2009) |
| *B. thuringiensis* strains |  |  |
| HD73 | Wild type containing *cry1Ac* gene | (Du and Nickerson, 1996; Liu et al., 2017) |
| HD (Δ*mclX*) | HD73 Δ*mclX* mutant | This study |
| HD (Δ*gerE*) | HD73 Δ*gerE* mutant | (Wang et al., 2009) |
| HD (Δ*sigK*) | HD73 Δ*sigK* mutant | (Du et al., 2011) |
| HD (P*mclX*-lacZ) | HD73 strain containing plasmid pHT P*mclX* | This study |
| HDΔ*gerE* (P*mclX*-*lacZ*) | Δ*gerE* mutant containing plasmid pHT P*mclX* | This study |
| HDΔ*sigK* (P*mclX*-*lacZ*) | Δ*sigK* mutant containing plasmid pHT P*mclX* | This study |
| HD (Δ*mclX:: mclX*) | HD(Δ*mclX*) strain carrying pHTHF*mclX* | This study |
| HDΔ*mclX* (P*cwlC*-*lacZ*) | Δ*mclX* mutant containing plasmid pHT (P*cwlC*-*lacZ*) | This study |
| HD (P*cwlC*-*lacZ*) | HD73 containing plasmid pHT (P*cwlC*-*lacZ*) | (Chen et al., 2018) |
| HD (P*sigK*-*lacZ*) | HD73 containing plasmid pHT (P*sigK*-*lacZ*) | This study |
| HDΔ*mclX* (P*sigK*-*lacZ*) | Δ*mclX* mutant containing plasmid pHT (P*sigK*-*lacZ*) | This study |
| HD (P*gerE*-*lacZ*) | HD73 containing plasmid pHT HD (P*gerE*-*lacZ*) | This study |
| HDΔ*mclX* (P*gerE*-*lacZ*) | Δ*mclX* mutant containing plasmid pHT (P*gerE* -*lacZ*) | This study |
| Bti (Δ*cwlC*) | Bti Δ*cwlC* mutant | (Huang et al., 2021) |
| Bti (Δ*mclX*) | Bti Δ*mclX* mutant | This study |
| HD (P*bxpB*-*lacZ*) | HD73 containing plasmid pHT (P*bxpB*-*lacZ*) | This study |
| HDΔ*mclX* (P*bxpB*-*lacZ*) | Δ*mclX* mutant containing plasmid pHT (P*bxpB*-*lacZ*) | This study |

**Supplementary Table S2.** The primers used in this work.

| Primer | Sequence (5’-3’) |
| --- | --- |
| P*mclX*-F | AACTGCAGTAGTTCATACTTAGAGTTGAGCGGA |
| P*mclX*-R | CGGGATCCCTCTATCTCTCCTTTTTTTGACGTC |
| HF*mclX*-F | AACTGCAGTAGTTCATACTTAGAGTTGAGCGGA |
| HF*mclX*-R | CGGGATCCCTATTTTTTCTCTGTCAATTTAACTAAATAA |
| MclX-F | CGGGGATCCATGGCAAGAAAAAAACAACCTAA |
| MclX-R | GCGTCGACTTTTTTCTCTGTCAATTTAACTAAATAATT |
| *mclX*-1 | AGATCTATCGATGCATGCCATGGTACCCGGGAGGACACCATGTATTTCTTGTTCCTCG |
| *mclX*-2 | CTATCACCTCAAATGGTTCGCTGTTTTCTTGCCATCTCTATCTCTCCTT |
| *mclX*-3 | AAGGAGAGATAGAGATGGCAAGAAAACAGCGAACCATTTGAGGTGATAG |
| *mclX*-4 | GTTGTTGTTCAATTTATTCTCTCTTTCTAGAAATTCCTCGTAGGCGCTCG |
| *mclX*-5 | CGAGCGCCTACGAGGAATTTCTAGAAAGAGAGAATAAATTGAACAACAAC |
| *mclX*-6 | GTCTGCAGAAGCTTCTAGAATTCGAGCTCCGCCCTCTACGACGATAATCACTAC |
| P*cwlC*-F | CTTTTTTGTTCGTGTCGTCATT |
| P*cwlC*-R(FAM) | TATTCATCAACATCTCCTTTCTTAATA |

**Supplementary Table S3.** Plasmids used in this work.

| Plasmid |  |  |
| --- | --- | --- |
| pHT304-18Z | Promoterless *lacZ* vector, Ery^r^, Amp^r^, 9.7 kb | (Hervé and Lereclus, 1994) |
| pET-21b | Expression vector, Amp^r^, 5.4kb | Novagen |
| pHT315 | *B. thuringiensis*-*E. coli* shuttle vector | (Arantes and Lereclus, 1991) |
| PMAD | Bt-*E. coli* shuttle, temperature-sensitive plasmid, Amp^r^, Ery^r^ | (Arnaud et al., 2004) |
| pHTP*mclX* | pHT304-18Z carrying P*mclX*, Amp^r^, Ery^r^ | This study |
| pET*mclX* | pET-21b containing *mclX* gene,Amp^r^ | This study |
| pHTHF*mclX* | pHT315 containing P*mclX*-*mclX* | This study |
| pHTP*cwlC* | pHT304-18Z carrying P*cwlC*, Amp^r^, Ery^r^ | (Chen et al., 2018) |
| pHTP*bxpB* | pHT304-18Z carrying P*bxpB*, Amp^r^, Ery^r^ | (Peng et al., 2016) |
| pHTP*sigK* | pHT304-18Z carrying P*sigK*, Amp^r^, Ery^r^ | This study |
| pHTP*gerE* | pHT304-18Z carrying P*gerE*, Amp^r^, Ery^r^ | This study |

**Supplementary Table S4.** High expression hypothetical proteins with unknown domain

| gene | T7 FPKM | annotation | domain |
| --- | --- | --- | --- |
| HD73_5208 | 1098.01 | hypothetical protein | No domain |
| HD73_4527 | 1108.91 | hypothetical protein | No domain |
| HD73_5409 | 1159.41 | hypothetical protein | DUF3920 family protein |
| HD73_1180 | 1184 | hypothetical protein | No domain |
| HD73_4208 | 1265.15 | hypothetical protein | DUF177 domain-containing protein |
| HD73_4852 | 1292.97 | hypothetical protein | YuzL family protein |
| HD73_1394 | 1339.26 | hypothetical protein | DUF3813 domain-containing protein |
| HD73_1166 | 1343.85 | hypothetical protein | DUF2639 domain-containing protein |
| HD73_4028 | 1388.45 | hypothetical protein | No domain |
| HD73_4534 | 1414.02 | hypothetical protein | No domain |
| HD73_1644 | 1593.74 | hypothetical protein | No domain |
| HD73_5643 | 1659.12 | hypothetical protein | No domain |
| HD73_5056 | 1712.9 | hypothetical protein | No domain |
| HD73_5375 | 1746.11 | hypothetical protein | YuzL family protein |
| HD73_2543 | 1883.31 | hypothetical protein | No domain |
| HD73_0343 | 1889.53 | hypothetical protein | Toxin-antitoxin system HicB family |
| HD73_0893 | 2003.77 | hypothetical protein | YhdB-like protein |
| HD73_1407 | 2004.94 | hypothetical protein | DUF3603 family protein |
| HD73_3382 | 2102.83 | hypothetical protein | No domain |
| HD73_4592 | 2115.92 | hypothetical protein | VrrA protein |
| HD73_2545 | 2175.45 | hypothetical protein | YozD family protein |
| HD73_4428 | 2201.59 | hypothetical protein | No domain |
| HD73_1625 | 2355.67 | hypothetical protein | DUF3911 domain-containing protein |
| HD73_3478 | 2389.99 | hypothetical protein | No domain |
| HD73_4243 | 2589.48 | hypothetical protein | No domain |
| HD73_0345 | 2847.61 | hypothetical protein | DUF2892 domain-containing protein |
| HD73_1802 | 2906.21 | hypothetical protein | No domain |
| HD73_2410 | 3054.8 | hypothetical protein | No domain |
| HD73_5096 | 3318.62 | hypothetical protein | No domain |
| HD73_5057 | 3695.97 | hypothetical protein | DUF2524 family protein |
| HD73_4703 | 4834.34 | hypothetical protein | No domain |
| HD73_1729 | 6583.09 | hypothetical protein | YpzI family protein |
| HD73_2829 | 7380.19 | hypothetical protein | No domain |
| HD73_5046 | 8023.98 | hypothetical protein | No domain |
| HD73_3828 | 8137.13 | hypothetical protein | YfhD family protein |
| HD73_4070 | 11340.2 | hypothetical protein | DUF3243 domain-containing protein |
| HD73_1451 | 11370.4 | hypothetical protein | No domain |
| HD73_5537 | 12290.43 | hypothetical protein | DUF1657 domain-containing protein |
| HD73_0587 | 19058.02 | hypothetical protein | YpzG family protein |
| HD73_5368 | 23415.04 | hypothetical protein | No domain |

**References**

Arantes, O. and Lereclus, D. (1991). Construction of cloning vectors for *Bacillus thuringiensis*. *Gene* 108, 115-119.

Arnaud, M. and Chastanet, A. (2004). Debarbouille, M. New vector for efficient allelic replacement in naturally nontransformable, low-GC-content, gram-positive bacteria. *Appl. Environ. Microbiol.* 70, 6887-6891.

Chen, X., Gao, T., Peng, Q., Zhang, J., Chai, Y., and Song, F. (2018). Novel cell wall hydrolase CwlC from *Bacillus thuringiensis* is essential for mother cell lysis. *Appl. Environ. Microb.* 84, e02640-02617.

Du, C. and Nickerson, K. W. (1996). *Bacillus thuringiensis* HD73 spores have surface localized Cry1Ac toxin: physiological and pathogenic consequences. *Appl. Environ. Microb.* 62, 3722-3726.

Du, L., Wei, J., Han, L., Zhen, C., Zhang, J., and Song, F. (2011). Characterization of *Bacillus thuringiensis* *sigK* disruption mutant and its influence on activation of *cry3A* promoter. *Wei sheng wu xue bao* 51, 1177-1184.

Foster, S. J. (1992). Analysis of the autolysins of *Bacillus subtilis* 168 during vegetative growth and differentiation by using renaturing polyacrylamide gel electrophoresis. *J. Bacteriol.* 174, 464-470.

Hervé, A. and Lereclus, D. (1994). Structural and functional analysis of the promoter region involved in full expression of the *cryIIIA* toxin gene of *Bacillus thuringiensis*. *Mol. Microbiol.* 13, 97-107.

Huang, L., Xu, L., Han, G., Crickmore, N., Song, F., and Xu, J. (2021). Characterization of CwlC, an autolysin, and its role in mother cell lysis of *Bacillus thuringiensis* subsp. *israelensis*. *Lett. Appl. Microbiol.* 74, 92-102. doi: 10.1111/lam.13590

Liu, G., Lai, S., Shu, C., Wang, P., and Song, F. (2017). Complete Genome Sequence of *Bacillus thuringiensis* subsp. *kurstaki* Strain HD73. *Genome Announc.* 1, e0008013.

Peng, Q., Kao, G., Qu, N., Zhang, J., Li, J., and Song, F. (2016). The Regulation of exosporium-related genes in *Bacillus thuringiensis*. *Sci. Rep.* 6, 19005.

Wang, G., Zhang, J., Song, F., Wu, J., Feng, S., and Huang, D. (2006). Engineered *Bacillus thuringiensis* GO33A with broad insecticidal activity against lepidopteran and coleopteran pests. *Appl. Microbiol. Biot.* 72, 924 –930.
